# Flavonoids and fagopyrins in buckwheat leaves and flowers during the growing season and their relationship with weather conditions

**DOI:** 10.1515/biol-2025-1280

**Published:** 2026-02-24

**Authors:** Eva Tavčar, Nina Kočevar Glavač, Marko Vidak

**Affiliations:** Department of Pharmaceutical Biology, Faculty of Pharmacy, University of Ljubljana, Aškerčeva cesta 7, SI-1000, Ljubljana, Slovenia; Institute of Biostatistics and Medical Informatics, Faculty of Medicine, University of Ljubljana, Vrazov trg 2, SI-1000, Ljubljana, Slovenia

**Keywords:** *Fagopyrum esculentum*, phenolic compounds, antioxidant activity, meteorology, cultivation

## Abstract

This study quantified how the concentrations of antioxidant flavonoids (particularly rutin and quercitrin), antioxidant activities, and phototoxic fagopyrin concentrations in buckwheat leaves and flowers varied during the growing season. In addition, the impact of meteorological variables (high and low temperature, sunshine, and precipitation) on these variations was investigated using pairwise Pearson correlation coefficients and corresponding false discovery rate (FDR)-adjusted *p*-values. The rutin concentration was 1.5-times higher, the antioxidant activity measured by the reducing power (RP) method was 5.1-times higher, and the fagopyrin concentration was 12.7-times higher in buckwheat flowers than in leaves during the peak flowering period. After peak flowering, the concentrations of phenolic compounds (but not fagopyrins) and antioxidant activities in the flowers sharply decreased. During 4-day dry periods, the RP antioxidant activity in buckwheat flowers was significantly positively correlated with both the average maximum temperature and the average minimum temperature. Weather conditions had no effect on fagopyrin accumulation in the flowers, but during dry periods there was a significant positive correlation between the average low temperature and fagopyrin concentration in the leaves. Although the observed correlations do not imply causation, they could be relevant for cultivation strategies to maximize the antioxidant capacity of harvested plant parts.

## 1 Introduction

Common buckwheat (*Fagopyrum esculentum* Moench) is a nutritionally and commercially important pseudocereal that contains antioxidant phenolic compounds, such as flavonoids like rutin and quercitrin, which have potential anti-inflammatory and cardiovascular protective effects [1], 2]. Previous studies have shown that the concentrations of these compounds vary between different buckwheat species and especially between different parts of the plant [3].

In addition to these beneficial flavonoids, buckwheat also produces fagopyrins, a class of phototoxic naphthodianthrones [4]. These secondary metabolites can cause photosensitization and skin irritation (fagopyrism); however, recent research in another species of the *Fagopyrum* genus (*Fagopyrum tataricum*) has highlighted their potential antimicrobial and anticancer activities [5], 6]. Although the general phytochemical profile of buckwheat is well characterized, there is a significant knowledge gap regarding fagopyrins, as precise analytical methods for their detection have only recently been developed. While the biosynthesis of flavonoids in buckwheat has been widely studied, the relationship between fagopyrin accumulation and specific weather parameters remains unclear. Abiotic stress, such as exposure to toxic chemicals or extreme weather conditions, triggers defense mechanisms in plants that can alter the synthesis of phenolic compounds, flavonoids, and other secondary metabolites [7], 8].

We therefore investigated how the concentrations of phenolic compounds (total phenolic compounds, total flavonoids, rutin, quercitrin), fagopyrins, and total antioxidant activity in buckwheat leaves and flowers changed during the growth phase. We also analyzed the influence of meteorological variables (high and low temperature, precipitation, and sunshine duration) during the sampling periods on these parameters. In the statistical analysis, we distinguished between wet and dry sampling periods, as we hypothesized that temperature and drought stress modulate flavonoid and fagopyrin concentrations and antioxidant activity in buckwheat through different accumulation patterns in flowers and leaves.

## 2 Materials and methods

### 2.1 Plant material, sampling, and extraction

The Darja variety (*F. esculentum* Moench), grown from seeds obtained from the Faculty of Biotechnology (Ljubljana, Slovenia), was used for the cultivation trials. Sowing took place on July 4, 2015, in a field in Gameljne, central Slovenia (46°07′09.2″ N, 14°29′51.5″ E, elevation 310 m above sea level). Plant material was sampled at 14 time points at 4-day intervals between August 3 and September 24, 2015.

Three biological replicates of leaves and flowers were sampled at each time point. Thus, six samples (three leaves and three flowers) were collected at each time point. Each biological replicate consisted of leaves or flowers taken from 30 different individual plants randomly selected throughout the field. The collected plant material was dried and milled to a fine powder using a Waring Commercial 8010 EB blender (Stamford, CT, USA). For extraction, the same protocol was used for each biological replicate: 100 mg of the milled sample was weighed in triplicate into 15 mL plastic flasks and extracted with 10 mL of acetone:water (9:1, v/v) purchased from Panreac (Barcelona, Spain). The flasks were incubated for 2 h at 60 °C. The resulting extracts were filtered through a 0.22 µm syringe filter before all subsequent analyses.

### 2.2 Spectrophotometric analyses of antioxidant activities and phytochemical content

All spectrophotometric assays were performed in triplicate in 330-µL TPP microplates (Thermo Fisher Scientific, Waltham, MA, USA) using a Macherey-Nagel Nanocolor UV-VIS spectrophotometer (Düren, Germany) and a BioTek Synergy H4 microplate reader (Thermo Fisher Scientific). If the initial absorbance measurement at the wavelength specific to each method was outside the linearity range (0.1–1.0), the sample was diluted to bring the absorbance within the linearity range (ideally around 0.7), and this dilution was used for further absorbance measurements. The average of the three absorbance measurements was used as the result of each spectrophotometric assay.

Antioxidant activity (AA) was determined using three spectrophotometric methods: the 2,2-diphenyl-1-picrylhydrazyl (DPPH) assay, the 2,2′-azino-bis(3-ethylbenzothiazoline-6-sulfonic acid) (ABTS) assay, and the reducing power (RP) assay. The DPPH assay was performed according to the method of Brand-Williams et al. [9] using the Fluka DPPH reagent (Honeywell, Charlotte, NC, USA). To 9 µL of each sample, 225 µL of DPPH reagent was added. Absorbance was measured at 517 nm after 30 min.

The ABTS assay was performed following the method described by Re et al. [10]. The ABTS reagent was obtained from Sigma-Aldrich (Saint Louis, MO, USA). The ABTS radical cation was generated by reacting a 7 mM ABTS solution with a 140 mM ammonium persulfate (Sigma-Aldrich) solution to prepare the ABTS-persulfate solution. Eight microliters of sample were combined with 312 µL of the ABTS-persulfate solution and incubated for 6 min. Absorbance was then measured at 734 nm.

The reducing power (RP) was determined as described by Oyaizu [11] using K_3_Fe(CN)_6_ from Kemika (Zagreb, Croatia), trichloroacetic acid (TCA) from Merck (Darmstadt, Germany), and FeCl_3_·6H_2_O from Sigma-Aldrich. For each sample, 5 µL of the stock solution was combined with 54 µL of 0.1 mol/L phosphate buffer (pH 7.0) and 60 µL of 1 % K_3_Fe(CN)_6_ solution. The mixture was incubated at 50 °C for 20 min. Then, 30 µL of 10 % TCA and 150 µL of purified water were added. Initial absorbance (*A*1) was measured at 700 nm. Next, 5 µL of 0.1 % FeCl_3_ solution was added, and the absorbance at 700 nm was measured again (*A*2). AA was calculated using the equation:

$$\left({A2}_{\text{sample}}{-A1}_{\text{sample}}\right)\;/\;\left(A2_{\text{blank}}{-A1}_{\text{blank}}\right)\text{.}$$

The total phenolic content (TPC) was determined using the Folin-Ciocalteu (FC) method following the procedure of Singleton and Rossi [12]. TPC was expressed as mg gallic acid equivalents (GAE) per gram of dry weight (DW) (mg GAE/g DW), calculated using a gallic acid standard. The FC reagent and sodium carbonate were purchased from Merck, and the gallic acid for the control samples was purchased from Sigma-Aldrich. Briefly, 40 µL of extract was mixed with 150 µL of purified water and 10 µL of FC reagent. After 3 min, 20 µL of 20 % sodium carbonate (Na_2_CO_3_) solution was added, and the mixture was incubated for 60 min. Absorbance was then measured at 750 nm.

Total flavonoid content (TFC) was determined using the aluminum chloride (AlCl_3_) method described by Zhishen et al. [13] and expressed as mg rutin equivalents (RE) per gram of dry weight (mg RE/g DW), calculated using a rutin standard. The AlCl_3_ solution was prepared with AlCl_3_·6H_2_O purchased from Sigma-Aldrich and methanol from Carlo Erba Reagents (Val de Reuil, France). To 200 µL of extract, 20 µL of 5 % AlCl_3_ solution in methanol was added. After a 30-min incubation, absorbance was measured at 425 nm.

### 2.3 HPLC analysis

Analyses were performed on a Shimadzu Prominence high-performance liquid chromatography (HPLC) system (Shimadzu, Kyoto, Japan) using a modified method based on Tavčar et al. [4]. For separation, a Phenomenex Kinetex XB-C18 column (100 × 4.6 mm, 2.7 µm) from Phenomenex (Torrance, CA, USA) was used. Acetonitrile and water were purchased from Panreac, and TFA was obtained from Roth. Separation was carried out at 40 °C with an injection volume of 5 µL. The mobile phase consisted of (A) water with 5 % acetonitrile and 0.1 % trifluoroacetic acid (TFA), and (B) acetonitrile with 5 % water and 0.1 % TFA, applied with a gradient program at 2 mL/min. The gradient program was as follows: 0 % B (0–0.5 min), increasing to 49 % B (0.5–6.0 min), then to 100 % B (6.0–30.0 min), held at 100 % B (30.0–36.0 min), and returning to 0 % B (36.0–40.0 min). Rutin and quercitrin were detected using an SPD-M20A photodiode array detector (PDA) at 254 nm, while fagopyrins were detected using a Shimadzu RF-10A XL fluorescence detector (excitation wavelength 330 nm, emission wavelength 590 nm).

The linearity and limits of quantification (LOQ) of the method were validated by measuring the areas under the curve (AUC) of seven dilutions of respective reference standards. The rutin standard was purchased from Sigma-Aldrich, the quercitrin dihydrate standard from Roth (Karlsruhe, Germany), and the hypericin standard (used as the external standard for quantification of fagopyrins) from Sigma-Aldrich. Standards of quercitrin and hypericin were first dissolved to prepare 0.1 mg/mL stock solutions (i.e., the least diluted solution for the calibration curve), while the rutin stock solution had a concentration of 2.0 mg/mL. Further dilutions for the standard calibration curves were prepared from each of these stock solutions. The most diluted solutions reached the lower LOQs (0.003 mg/mL for rutin, 1.2 × 10^−5^ mg/mL for quercitrin, 1.23 × 10^−4^ mg/mL for hypericin). AUCs of analyzed samples were converted to concentrations using equations derived from the standard calibration curves with the coefficients of determination (*R* ^2^) of 0.997 for rutin, 1.000 for quercitrin, and 0.999 for hypericin. The concentrations of quercitrin were corrected for the dihydrate form of the reference standard using the equation:

$${\text{conc}}_{q}=\left(\text{con}c_{q-\text{dihydrate}\;}{\times M}_{q}\right)\;/\;M_{q-\text{dihydrate}}$$

where conc is concentration, q is quercetin, and *M* is molar mass.

### 2.4 Meteorological data

Meteorological data (high and low temperature, precipitation, and sunshine duration) for 2015 were obtained from the database of the Slovenian Environment Agency [14]. The data were recorded at the Ljubljana-Bežigrad weather station, located 8 km south of the buckwheat field. The raw data were summarized into 4-day intervals corresponding to the sampling periods. Four-day averages were calculated for both maximum and minimum temperatures, while 4-day totals were used for precipitation and sunshine duration. For these totals, only the precipitation and sunshine duration in the four days preceding each sampling were considered, not the cumulative sums from the first sampling onward. The intervals were classified as “wet” (W, total precipitation > 1 mm) or “dry” (D, total precipitation ≤ 1 mm; Table 1). The 1 mm threshold was used because this amount of precipitation is equivalent to one wet day according to the definition of the World Meteorological Organization [15].

**Table 1:** Sampling intervals.

Time interval (in 2015)	Precipitation [mm]	Interval number (D–dry, W–wet)
July 30 – August 2	51.4	W1
August 3 – August 6	0.1	D1
August 7 – August 10	0	D2
August 11 – August 14	0	D3
August 15 – August 18	15.8	W2
August 19 – August 22	33.9	W3
August 23 – August 26	40.4	W4
August 27 – August 30	0	D4
August 31 – September 3	0.7	D5
September 4 – September 7	86.3	W5
September 8 – September 11	0	D6
September 12 – September 15	1.4	W6
September 16 – September 19	7	W7
September 20 – September 23	0.8	D7

The meteorological variables were defined as follows:

– Average 4-day maximum temperature: sum of maximum temperatures in the four days preceding the sampling divided by the number of days (e.g., for the August 19 sampling, the sum of the maximum temperatures on August 15, 16, 17, and 18 was used, divided by 4).
– Average 4-day minimum temperature: sum of minimum temperatures in the four days preceding the sampling divided by the number of days.
– 4-day sum of precipitation: sum of precipitation in the four days preceding the sampling.
– 4-day sum of sunshine duration: sum of sunshine duration in the four days preceding the sampling.

### 2.5 Statistical analysis

The analysis used the following 12 variables (4 meteorological and 8 non-meteorological):

Average 4-day maximum temperature; average 4-day minimum temperature; 4-day sum of precipitation; 4-day sum of sunshine duration; antioxidant activity (AA) – DPPH method; AA – ABTS method; AA – reducing power method; concentration of total phenolic compounds – FC method; concentration of total flavonoids – AlCl_3_ method; concentration of rutin – HPLC; concentration of quercitrin – HPLC; concentration of fagopyrins – HPLC.

Pairwise Pearson correlation matrices were calculated in Excel (Microsoft, Redmond, WA, USA), resulting in 11 correlations tested for each variable. Separate pairwise correlation matrices for leaves and flowers were created for all, wet, and dry intervals. *p*-Values were calculated from the Pearson correlation coefficients using SPSS version 26 software (IBM, Armonk, NY, USA), and the Benjamini-Hochberg method [16] was used for false discovery rate (FDR) correction. Corrected *p*-values less than 0.05 were considered significant. The pairwise correlation matrices with all Pearson coefficients and corresponding *p*-values are available in an Excel worksheet in the Research data (Digital Commons Data dataset). The dataset also includes data values for all variables and time points.

## 3 Results

### 3.1 Weather conditions

Between July 30 and September 23, 2015, there were seven dry 4-day periods (D1–D7) with total precipitation per period of less than 1 mm, while the other seven 4-day periods were classified as wet (W1–W7) (Table 1).

**Figure 1:**
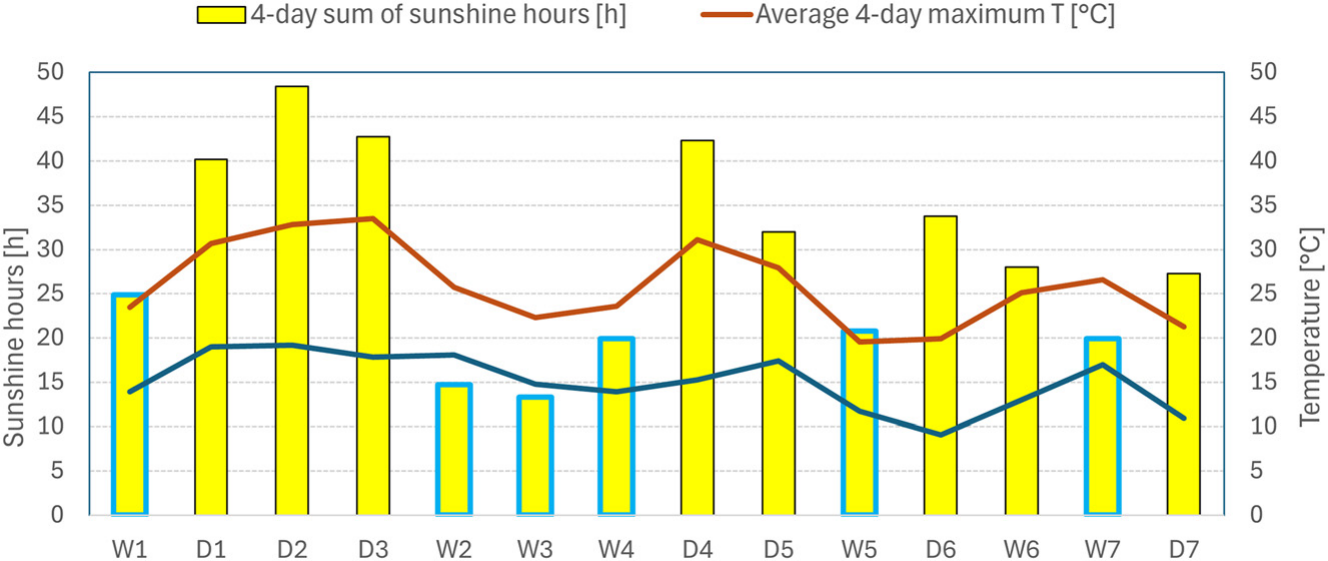
Weather conditions during the 2015 growing season. Notes: bars representing sunshine hours during wet periods are marked with a blue border. Labels: *T*, temperature; h, hours; D1–D7, dry sampling periods; W1–W7, wet sampling periods.

The wet periods included the first sampling period (July 30 – August 2), during which only leaf samples were collected because the plants were not yet flowering. Therefore, the statistical analysis for leaves across all periods was based on 14 time points (*n* = 14); for flowers across all periods, *n* = 13; for both leaves and flowers in dry periods, *n* = 7; for leaves in wet periods, *n* = 7; and for flowers in wet periods, *n* = 6. Meteorological data (temperature, precipitation, sunshine) for the 14 sampling intervals are presented in Figure 1.

### 3.2 Concentration of secondary metabolites

**Figure 2:**
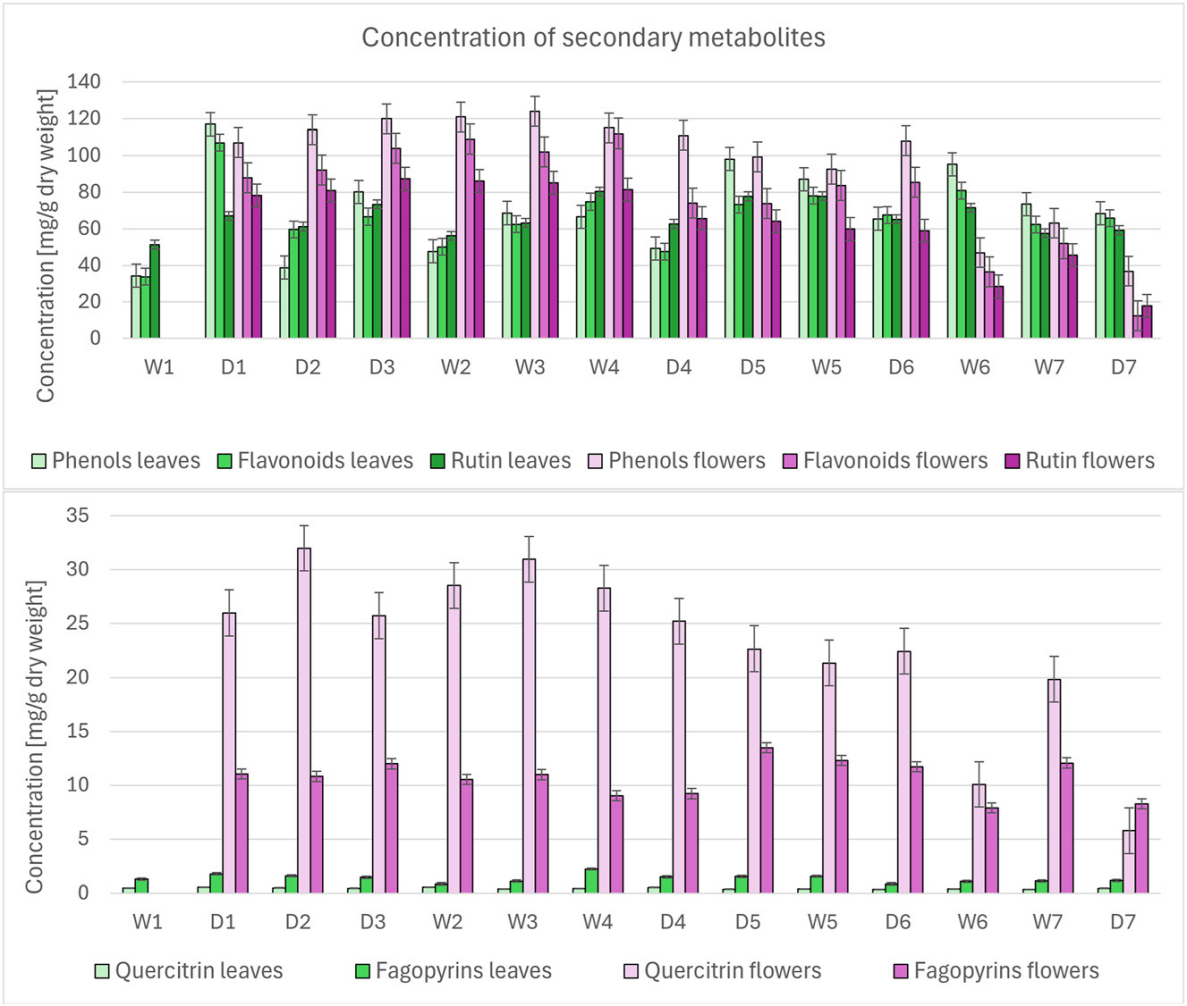
Concentrations of secondary metabolites in buckwheat leaves and flowers. Notes: concentrations are expressed in mg substance/g dried plant material (dry weight). Concentrations of rutin, quercitrin, and fagopyrins were measured with high-performance liquid chromatography (HPLC). Error bars represent standard error. Labels: phenols, concentration of total phenolic compounds – FC method; flavonoids, concentration of total flavonoids – AlCl_3_ method; D1–D7, dry sampling periods; W1–W7, wet sampling periods.

Analysis of the phytochemical content revealed that buckwheat flowers contained more secondary metabolites than the leaves during the main flowering period (approximately August 7 to August 30), but the concentrations of phenolic compounds in the flowers decreased rapidly after this period (Figure 2).

The concentration of the most abundant flavonoid, rutin, in the flowers decreased 3.7-fold from 66 mg/g dry weight (DW) at the end of August to 18 mg/g DW in the last sampling period (September 20–23). In contrast, the concentration of fagopyrins in the flowers remained constant across all sampling periods and did not decrease sharply in September. The concentration of the second most abundant flavonoid, quercitrin, in the flowers reached about a quarter of the rutin concentration during the main flowering period, while in the leaves, the quercitrin concentration was approximately 100-fold lower than the rutin concentration throughout August and September.

### 3.3 Antioxidant activity

**Figure 3:**
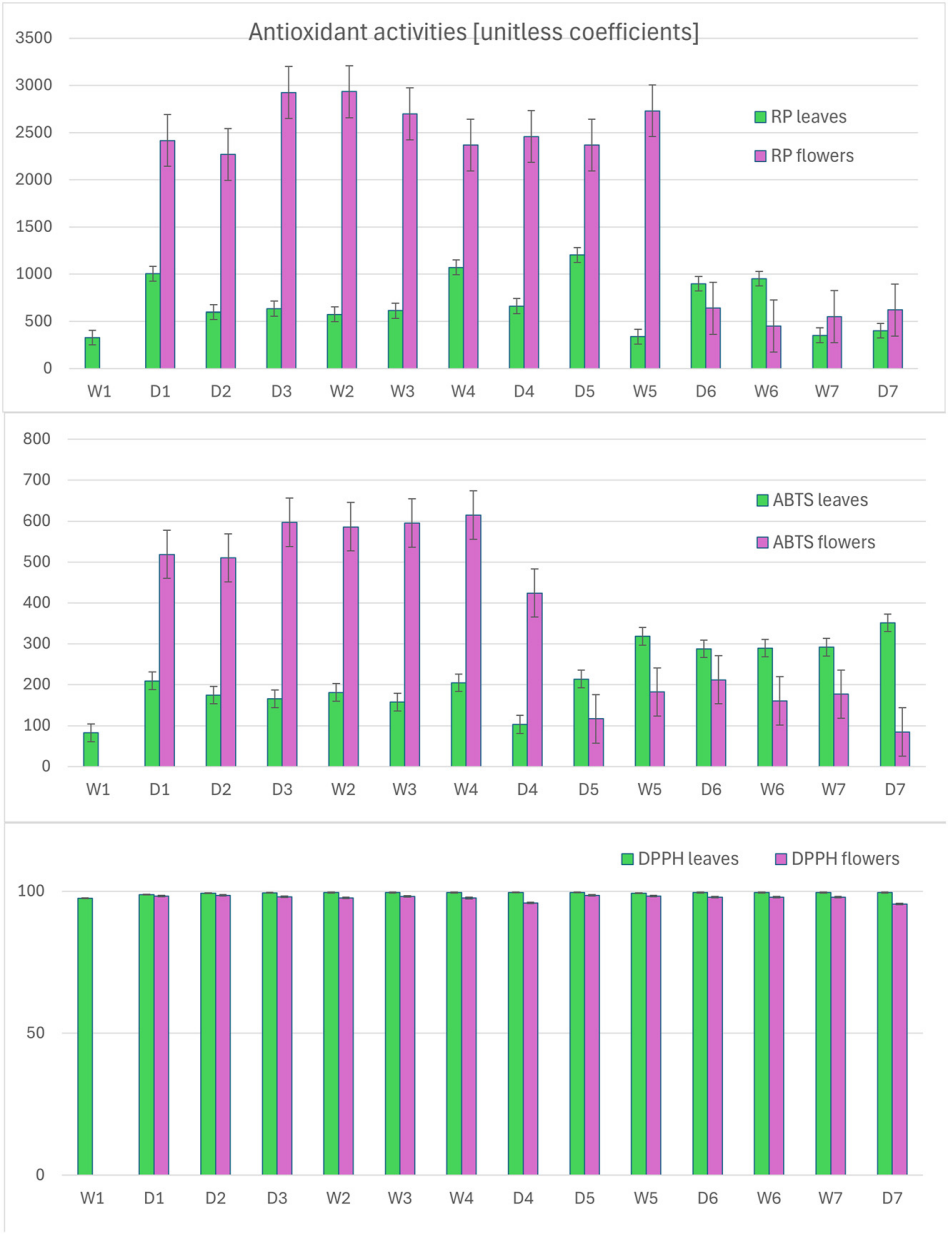
Antioxidant activity in buckwheat leaves and flowers. Notes: error bars represent standard error. Antioxidant activity coefficients for different methods are not linearly related and therefore are not directly comparable. Labels: RP, reducing power method; ABTS, 2,2′-azino-bis(3-ethylbenzothiazoline-6-sulfonic acid) method; DPPH, 2,2-Diphenyl-1-picrylhydrazyl method; D1–D7, dry sampling periods; W1–W7, wet sampling periods.

The ABTS and RP assays showed a peak in antioxidant potential in the flowers during intense flowering, followed by a sharp decline, while AA in the leaves generally remained constant (Figure 3).

These results in the flowers reflect the trends observed for phenolic compounds. According to both the ABTS and RP methods, AA in the flowers decreased by approximately 4- to 5-fold between August 30 and September 23.

### 3.4 Correlations between the meteorological and non-meteorological variables

Correlations between the meteorological and non-meteorological variables, such as concentrations of secondary metabolites and antioxidant activities, are shown in Table 2 as Pearson correlation coefficients (*r*). *p*-Values (both unadjusted and FDR-adjusted) are included when unadjusted *p*-values were below 0.05. FDR-corrected *p*-values less than 0.05 were considered significant.

**Table 2:** Pearson correlation coefficients between the meteorological and non-meteorological variables (antioxidant activity and concentrations of secondary metabolites), with *p*-values for correlations with unadjusted *p* < 0.05.

Other variables	Sample type	Meteorological variables			
		Minimum T	Maximum T	Precipitation	Sunshine
DPPH	All (L) *n* = 14	−0.027	0.048	0.36	−0.014
	Dry (L) *n* = 7	−0.48	−0.32	0.30	−0.34
	Wet (L) *n* = 7	0.20	0.14	−0.35	−0.41
	All (F) *n* = 13	0.39	0.13	0.21	−0.019
	Dry (F) *n* = 7	0.55	0.36	−0.31	0.40
	Wet (F) *n* = 6	−0.49	−0.70	0.55	−0.068
ABTS	All (L) *n* = 14	−0.49	−0.50	−0.014	−0.21
	Dry (L) *n* = 7	−0.68	−0.86* *p* = 0.012 FDRp = 0.052	0.61	−0.80* *p* = 0.029 FDRp = 0.11
	Wet (L) *n* = 7	−0.23	−0.045	−0.055	0.22
	All (F) *n* = 13	0.52	0.43	0.048	0.063
	Dry (F) *n* = 7	0.67	0.83* *p* = 0.021 FDRp = 0.058	−0.80* *p* = 0.031 FDRp = 0.096	0.90* *p* = 0.0053 FDRp = 0.058
	Wet (F) *n* = 6	0.36	0.0022	0.0038	−0.73
RP	All (L) *n* = 14	0.13	0.20	−0.39	0.24
	Dry (L) *n* = 7	0.24	0.016	0.051	−0.13
	Wet (L) *n* = 7	−0.15	0.19	−0.37	0.14
	All (F) *n* = 13	0.52	0.37	0.37	0.051
	Dry (F) *n* = 7	0.90** *p* = 0.0053 **FDRp = 0.029**	0.95** *p* = 0.00083 **FDRp = 0.0091**	−0.37	0.69
	Wet (F) *n* = 6	0.00075	−0.57	0.63	−0.72
Phenols	All (L) *n* = 14	0.058	0.033	−0.12	0.049
	Dry (L) *n* = 7	0.26	0.0089	0.29	−0.33
	Wet (L) *n* = 7	−0.47	−0.19	−0.070	0.26
	All (F) *n* = 13	0.40	0.32	0.15	0.14
	Dry (F) *n* = 7	0.52	0.64	−0.81* *p* = 0.028 FDRp = 0.096	0.76* *p* = 0.046 FDRp = 0.085
	Wet (F) *n* = 6	0.23	−0.33	0.38	−0.86* *p* = 0.030 FDRp = 0.11
Flavonoids	All (L) *n* = 14	0.061	0.047	−0.12	0.15
	Dry (L) *n* = 7	0.29	0.016	0.081	−0.13
	Wet (L) *n* = 7	−0.48	−0.25	0.0093	0.16
	All (F) *n* = 13	0.39	0.22	0.30	−0.020
	Dry (F) *n* = 7	0.55	0.62	−0.79* *p* = 0.035 FDRp = 0.096	0.74
	Wet (F) *n* = 6	0.19	−0.33	0.42	−0.77
Rutin	All (L) *n* = 14	−0.085	−0.0021	0.18	0.081
	Dry (L) *n* = 7	0.39	0.27	0.13	−0.078
	Wet (L) *n* = 7	−0.65	−0.50	0.34	0.15
	All (F) *n* = 13	0.57* *p* = 0.041 FDRp = 0.15	0.42	0.16	0.10
	Dry (F) *n* = 7	0.74	0.80* *p* = 0.032 FDRp = 0.070	−0.74	0.83* *p* = 0.021 FDRp = 0.059
	Wet (F) *n* = 6	0.32	−0.24	0.31	−0.88* *p* = 0.022 FDRp = 0.11
Quercitrin	All (L) *n* = 14	0.53	0.54* *p* = 0.048 FDRp = 0.13	−0.19	0.33
	Dry (L) *n* = 7	0.54	0.64	−0.37	0.60
	Wet (L) *n* = 7	0.50	0.26	−0.067	−0.27
	All (F) *n* = 13	0.55	0.38	0.16	0.098
	Dry (F) *n* = 7	0.67	0.72	−0.76* *p* = 0.045 FDRp = 0.099	0.87* *p* = 0.011 FDRp = 0.059
	Wet (F) *n* = 6	0.37	−0.21	0.30	−0.92* *p* = 0.0082 FDRp = 0.090
Fagopyrins	All (L) *n* = 14	0.28	0.33	0.22	0.29
	Dry (L) *n* = 7	0.95** *p* = 0.0010 **FDRp = 0.011**	0.86* *p* = 0.014 FDRp = 0.052	−0.12	0.57
	Wet (L) *n* = 7	−0.50	−0.41	0.49	0.14
	All (F) *n* = 13	0.30	0.11	0.15	0.05
	Dry (F) *n* = 7	0.35	0.20	−0.086	0.082
	Wet (F) *n* = 6	0.19	−0.31	0.47	−0.51

Unadjusted *p*-values and false discovery rate (FDR)-adjusted *p*-values (FDRp) are shown for correlations with unadjusted *p* < 0.05. Labels: minimum T, average 4-day minimum temperature; maximum T, average 4-day maximum temperature; precipitation, 4-day sum of precipitation; sunshine, 4-day sum of sunshine duration; DPPH, antioxidant activity (AA) – DPPH method; ABTS, AA – ABTS method; RP, AA – reducing power method; phenols, concentration of total phenolic compounds – FC method; flavonoids, concentration of total flavonoids – AlCl_3_ method; rutin, concentration of rutin – HPLC; quercitrin, concentration of quercitrin – HPLC; fagopyrins, concentration of fagopyrins – HPLC; (L), buckwheat leaves; (F), buckwheat flowers; all, all periods; dry: dry periods (precipitation < 1 mm); wet, wet periods; *n*, number of measurements (time points); FDRp, false discovery rate-adjusted *p*-value; *, unadjusted *p*-value < 0.05; **, FDR-adjusted *p*-value < 0.05 (significant correlation). Bold values indicate correlation coefficients and FDR-adjusted *p*-values for significant correlations.

**Figure 4:**
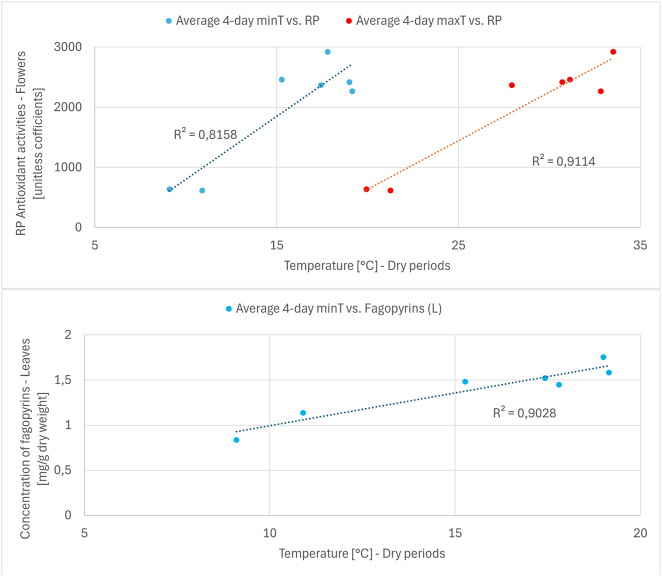
Observed significant correlations (FDR-adjusted *p* < 0.05) between the meteorological and non-meteorological variables (antioxidant activities and concentrations of secondary metabolites). Labels: minT, minimum temperature; maxT, maximum temperature; RP, antioxidant activity – reducing power method; fagopyrins (L), concentration of fagopyrins in buckwheat leaves – HPLC method; *R* ^2^, coefficient of determination.

In buckwheat leaves, there was a strong and significant positive correlation during dry periods between fagopyrin concentration and average minimum temperature (*r* = 0.95, FDR-adjusted *p* = 0.011, *n* = 7). Under higher minimum nighttime temperatures, which are common during summer heatwaves, fagopyrin concentration in the leaves increased. In buckwheat flowers, antioxidant activity (RP method) showed a significant positive correlation with both average maximum temperature (*r* = 0.95, FDR-adjusted *p* = 0.0091, *n* = 7) and average minimum temperature (*r* = 0.90, FDR-adjusted *p* = 0.029, *n* = 7) during dry periods, but the positive correlation with sunshine did not reach significance (*r* = 0.73, FDR-adjusted *p* = 0.15). Thus, during summer heatwaves, the antioxidant activity of the flowers increased. Other correlations in leaves and flowers, regardless of the period, were not significant when the FDR correction was applied. Figure 4 shows scatter plots for the observed significant correlations between the meteorological and non-meteorological variables.

Four-day sunshine duration showed no correlation with rutin (*r* = 0.10) or quercitrin (*r* = 0.10) in buckwheat flowers across all periods. However, when dry and wet periods were analyzed separately, strong positive correlations were observed during dry periods (rutin: *r* = 0.83; quercitrin: *r* = 0.87), and strong negative correlations during wet periods (rutin: *r* = −0.88; quercitrin: *r* = −0.92) (Table 2). These correlations do not imply causation, and they were no longer significant at the 0.05 level after FDR correction (e.g., FDR-adjusted *p* = 0.059 for the correlations of both rutin and quercitrin with sunshine duration in buckwheat flowers in dry periods), although the unadjusted *p*-values were below the 0.05 threshold (e.g., unadjusted *p*-values for the correlation with sunshine duration in flowers in dry periods were 0.021 for rutin and 0.011 for quercitrin).

## 4 Discussion

The 2015 growing season in the Ljubljana area was generally hot. For example, in July 2015, during the buckwheat growth phase before the first sampling, the maximum temperature exceeded 35 °C on eight days, and the minimum temperature exceeded 20 °C on 10 days. However, heatwaves with sunny, dry weather were interrupted by cold snaps that brought abundant rainfall and reduced, though not zero, sunshine. For instance, the maximum temperature dropped from 35 °C on August 13–20 °C on August 17. From mid-August, a general decrease in temperature and hours of sunshine was observed, corresponding to the seasonal transition from summer to early fall. Nevertheless, the maximum temperature reached 30 °C as late as September 17, while the minimum temperature dropped to 7 °C on September 22 [14].

Summer heatwaves feature sunny days with a very high midday ultraviolet (UV) radiation index, typically around 9 at the buckwheat cultivation site. UV rays can cause harmful oxidative stress in plants, which antioxidants such as flavonoids and other phenolic compounds can mitigate. The marked accumulation of these compounds in the flowers during peak flowering suggests that they play a primary role in protecting the plant’s sensitive reproductive organs from oxidative stress.

Regarding flavonoids, it is noteworthy that the HPLC-quantified rutin concentrations were in some cases higher than the total flavonoid content determined by the spectrophotometric AlCl_3_ assay. This is not unexpected, as spectrophotometric methods are less specific than HPLC. Their accuracy can be affected by the presence of other compounds that can chelate aluminum or by the different reactivity of various flavonoids, leading to an underestimation of the total content when calibrated against a single standard such as rutin [17]. This highlights the importance of using specific methods such as HPLC for accurate quantification of individual phenolic compounds.

Antioxidant potential in buckwheat flowers during dry periods (measured by the ABTS and RP methods) generally reflects the trends observed for rutin – the correlation with temperature and sunshine is positive, although it is rarely significant at the 0.05 level due to the FDR correction of unadjusted *p*-values (Table 2). The results of the DPPH assays, which differed from the ABTS and RP methods by showing minimal AA fluctuations in both leaves and flowers, likely indicate a limitation of this method for complex plant extracts, a phenomenon also reported in other studies [18]. As the most abundant flavonoid in buckwheat, rutin is probably responsible for most of the plant’s antioxidant potential. The higher AA in flowers is likely due to their higher concentrations of flavonoids, which are potent antioxidants [19].

The most interesting result was the reversal of the correlation between sunshine and flavonoid concentration during wet periods compared to dry periods. During the dry 4-day periods, hours of sunshine correlated positively with concentrations of rutin and quercitrin (Table 2), and the FDR-adjusted *p*-values were only marginally above the 0.05 significance threshold (FDR-adjusted *p* = 0.059 for both rutin and quercitrin). This aligns with the known role of flavonoids as UV-protective compounds, whose synthesis is upregulated in response to high light intensity and drought stress to protect the plant from photodamage [20]. However, during humid intervals, the correlation of sunshine duration with flavonoid concentration was negative but not significant at the 0.05 level (e.g., FDR-adjusted *p*-values were 0.11 and 0.090 for rutin and quercitrin, respectively). More sunshine during otherwise wet periods, such as changeable weather with frequent rain showers separated by short sunny intervals, correlated with lower concentrations of rutin and quercitrin.

We hypothesize that a water film on the flower surface may alter UV light intensity, possibly by reflecting or scattering some of the UV radiation and thereby reducing radiation stress. Alternatively, the water could lower the surface temperature of the petals, mitigating heat stress that might otherwise act synergistically with high light intensity to induce flavonoid production. The effect of moisture may be enhanced by trace minerals in rainwater, which have been shown to influence antioxidant activity in buckwheat sprouts [21], although this effect likely varies among different plant parts. Therefore, maximizing sunlight exposure alone is not sufficient to optimize yield. Just as mineral supplementation has been shown to alter rutin content in seeds [22], controlling water and light exposure can increase flavonoid content in flower biomass.

Temperature also played an important role. In leaves, fagopyrin concentration was positively correlated with summer heat waves when both high and low temperatures were elevated, which corresponds to dry periods in this analysis. The correlation of fagopyrin concentration in leaves with minimum temperature in dry periods was significant (Figure 4). This suggests that fagopyrins, like other secondary metabolites, may be part of a general stress response, with their production stimulated by temperature extremes. This is consistent with studies on the structurally similar compound hypericin. In flowers, higher temperatures during dry periods were significantly correlated with increased antioxidant activity (Figure 4), which was in turn correlated with higher rutin concentrations. This suggests that buckwheat flowers enhance their protective antioxidant systems under combined heat and drought stress, with rutin making an important contribution.

Plant genotype has been identified as the key factor in drought stress response. Drought stress can modify and regulate the expression of genes that encode important enzymes in the phenylpropanoid biosynthetic pathway [23]. This pathway branches into two, producing numerous metabolites related to lignin and flavonoids [24]. Thus, drought stress increases the production of phenolic compounds and flavonoids. However, the timeline of drought response may differ among various phenolic compounds within the same plant. Zhang et al. [25] examined the effect of drought stress on plant metabolism in *F. tataricum* seedlings and reported distinct accumulation patterns of phenylpropanoids (including flavonoids) after 7 and 11 days of drought exposure. For common buckwheat leaves, Sytar et al. [26] reported that rutin content increased early during drought stress, while chlorogenic acid and kaempferol increased only under severe dehydration. While our study did not observe an effect of short-term drought on the accumulation of phenolic compounds in buckwheat leaves, we did observe an increase in these compounds in buckwheat flowers during 4-day dry periods.

In the leaves, we observed an accumulation of fagopyrins during dry periods. Fagopyrins are structurally similar to hypericin from *Hypericum perforatum* (St. John’s wort), and the metabolism of this compound is affected by temperature [27], 28]. The study by Amooaghaie et al. [29] on *H. perforatum* flowering tops (containing flowers, leaves, and stems) reported an increase in hypericin concentration during 10-day drought intervals, but the concentration declined after 13 days of drought stress. Makarova et al. [30] noted the dependency of *H. perforatum* phenolic and flavonoid content on harvest time for *H. perforatum* cultivated under uncontrolled conditions in central Poland in a humid continental climate broadly similar to the climatic conditions in our study, though with less summer heat. The authors hypothesized that the relationship between phenolic content and harvest time could be correlated with the average maximum daily temperatures.

Although this field study provides data from buckwheat cultivation that replicated agricultural methods, it includes data from only a single growing season and one location. Therefore, interannual variability and genetic factors remain unaddressed, and the influence of annual fluctuations on the phytochemical composition and antioxidant potential of the plant still needs investigation. The reported correlations are observational and do not demonstrate a causal effect of weather on secondary metabolites and antioxidant activity. Future experiments in controlled greenhouses are needed to establish possible causation between growing conditions and the observed patterns of plant activity and metabolite accumulation. Additionally, statistical analysis in further studies could be strengthened by using multiple regression or principal component analysis.

## 5 Conclusions

This is the first study to examine the correlations between meteorological variables and the concentrations of phenolic compounds, fagopyrins, and antioxidant potential in common buckwheat. Significant correlations were found between elevated daytime and nighttime temperatures during dry 4-day periods and increased antioxidant capacity in buckwheat flowers, while high nighttime temperatures in dry periods were significantly correlated with higher fagopyrin concentrations in buckwheat leaves. Although this study was limited to one growing season and one location, the significant correlations observed indicate that the plant’s phytochemical composition and antioxidant potential are dynamic and change in response to meteorological conditions, particularly during summer droughts and heatwaves when both maximum and minimum temperatures are elevated. A deeper understanding of environmental response mechanisms could inform the development of cultivation and breeding strategies to maximize the antioxidant potential and flavonoid content of common buckwheat, such as cultivating drought-resistant varieties in drier climates or soils and harvesting flowers at peak bloom for flavonoid extraction. Until then, harvest dates could be selected based on weather patterns in the weeks preceding the planned harvest.
